# Effects of Extraoral Suction on Droplets and Aerosols for Infection Control Practices

**DOI:** 10.3390/dj9070080

**Published:** 2021-07-07

**Authors:** Hidenobu Senpuku, Masahiko Fukumoto, Toshikazu Uchiyama, Chieko Taguchi, Itaru Suzuki, Kazumune Arikawa

**Affiliations:** 1 Department of Bacteriology I, National Institute of Infectious Diseases, Tokyo 162-8640, Japan; suzuki.itaru@nihon-u.ac.jp; 2 Department of Microbiology and Immunology, Nihon University School of Dentistry at Matsudo, Chiba 271-8587, Japan; 3 Department of Laboratory Medicine for Dentistry, Nihon University School of Dentistry at Matsudo, Chiba 271-8587, Japan; fukumoto.masahiko@nihon-u.ac.jp; 4 Department of Operative Dentistry, Nihon University School of Dentistry at Matsudo, Chiba 271-8587, Japan; uchiyama.toshikazu@nihon-u.ac.jp; 5 Department of Community Oral Health, Nihon University School of Dentistry at Matsudo, Chiba 271-8587, Japan; taguchi.chieko@nihon-u.ac.jp (C.T.); arikawa.kazumune@nihon-u.ac.jp (K.A.); 6 Department of Pediatric Dentistry, Nihon University School of Dentistry at Matsudo, Chiba 271-8587, Japan

**Keywords:** extraoral suction, oral suction, droplets, aerosols, COVID-19, infection control practices, dental care

## Abstract

Dental professionals are at increased risk of being infected with airborne pathogens such as SARS-CoV-2 because they are often exposed to droplets/aerosols production during dental treatment. To scientifically clear the effects of extraoral and oral suctions on the droplets and aerosols produced by dental treatments using an ultrasonic scaler was analyzed. The adenosine triphosphate and bacteria in droplets and aerosols produced during simulated scaling were quantitatively observed by reactions with luciferin/luciferase and incubation in culture plates to grow bacteria, respectively. The protection against spreading droplets and aerosols by oral and extraoral suctions was recognized, and the areas were limited to the left and posterior sides of the dental chair head when a right-handed dentist and dental hygienist performed scaling. Extraoral suction is a very useful tool for reducing the infection risk of COVID-19 in dental care, but the effective area is limited depending on physical characteristics of dentist and dental hygienist.

## 1. Introduction

Daily dental treatments produce droplets and aerosols, providing dental professionals and patients with a risk of infection [1]. Customary dental procedures that include the handpiece and the use of rotary instruments such as the high-speed turbine handpiece and the use of ultrasonic scalers for dental calculus removal are associated with the production of large quantities of droplets and aerosols from the saliva and blood of patients. The majority of dental treatment procedures expose dental professionals to droplets/aerosols [2,3,4,5]. SARS-CoV-2 transmission during dental treatments can therefore occur during uptake of aerosols/droplets from infected patients or direct contact with surface of the oral mucosa, oral fluids, and contaminated dental instruments and surfaces around dental chair [6,7,8]. In a report about physician death from COVID-19, 6% (16/254) of physicians who died from COVID-19 were dentists in addition to general practitioners and emergency room doctor, 43% (108/254) [9]. However, another paper from China reported that no dentists have died from COVID-19 contracted during patient encounters [5]. In Japan, outbreak of infected patients have not occurred until now in dental office [10]. The Centers for Disease Control and Prevention have developed infection control guidelines that apply in the dental health care setting based on universal and standard precautions in Japan. These guidelines were developed to control the infection of pathogens between patients and dental staff. Events such as the 2009 H1N1 pandemic and Japan’s Health Care Reform in 2008 may have highlighted to the patient’s growing concerns about infection control practices (ICPs). Detailed education about ICPs at Japanese Universities also address these growing concerns [11,12]. The transmitting infection to dental practitioners and students were associated with their perception of the inadequacy of standard infection control [13,14]. The risk of virus infection still pose a tremendous hazard during the second wave of SARS-CoV-2 infection, as there are many patients who are asymptomatic or paucisymptomatic patients for COVID-19 [15]. Many clinical recommendations for safe dental treatment for COVID-19 emergencies have been reported to limit SARS-Cov-2 infection between dentists and patients [15,16]. Extra high-volume suction devices in ICPs have been recommended for use in conjunction with regular suction for aerosols, fomites, and saliva [2,17]. Therefore, to clear behavior in effects of infection control on the patients, the effectiveness of extraoral and oral suctions to spreading droplets and aerosols was observed in dental treatment.

Extraoral and oral suctions are indicated as excellent machinery and tools to protect against viral and bacterial infections in droplets and aerosols during dental treatment [18,19]. However, the effects by suctions were physically assessed and biological significance could not be evaluated. In this study, the effects of extraoral and oral suction on droplets and aerosols were assessed by biological evaluation procedures in private rooms from dental offices and university dental school hospitals. The protection against spreading droplets and aerosols by the combination of extraoral and oral suctions was recognized and the protected areas were limited to the left and posterior sides of the dental chair head when a right-handed dentist and dental hygienist performed scaling. 

## 2. Materials and Methods

### 2.1. Setting of Simulated Scaling

**Figure 1:**
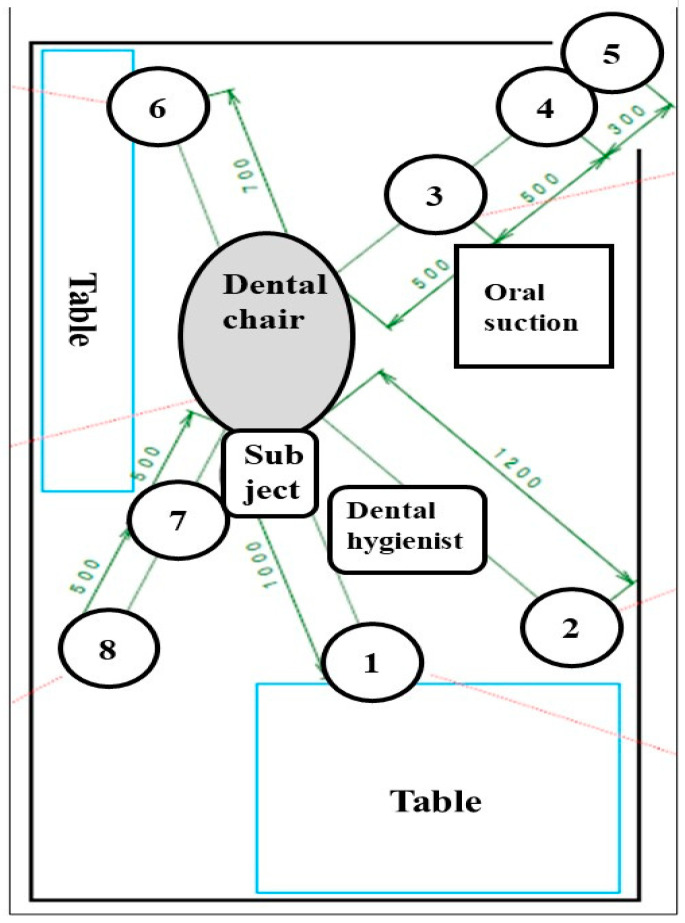
Sampling areas of droplets and aerosols for detection of bacterial numbers. Numbers (1~8) indicate the location of BHI agar and R2A agar plates that were placed radially from the dental chair at 1-m intervals.

**Figure 2:**
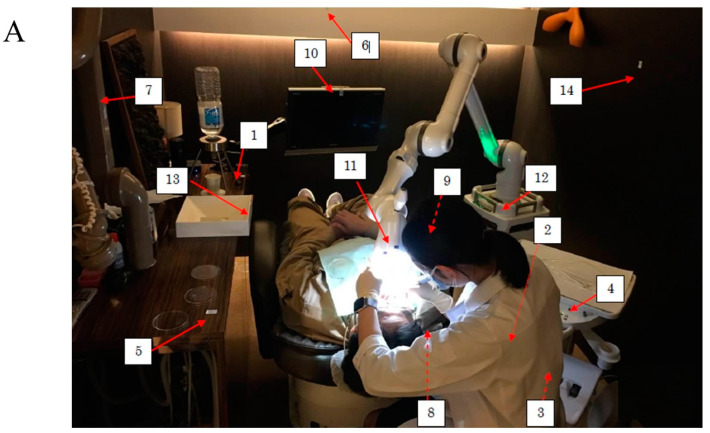

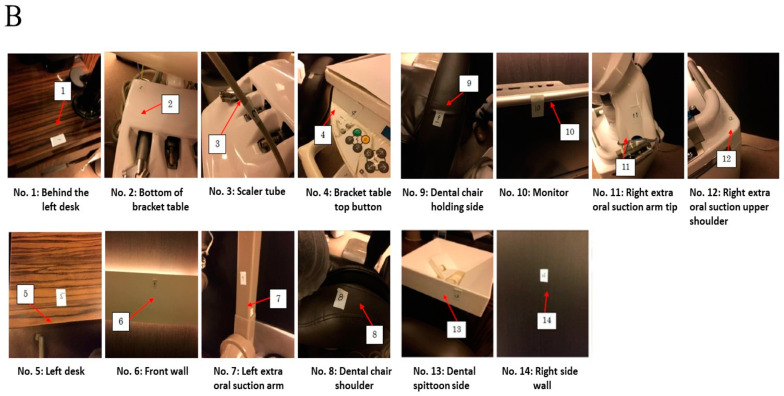
Sampling areas of droplets and aerosols for the detection of RLU. Numbers (1~14) in the picture indicate the locations of sampling areas that were swabbed by the LuciPac pen around the dental chair. (**A**): Whole photo, (**B**): Photographs of individual parts. A volunteer provided consent to share the picture.

Private rooms in a private dental office (AO1 Dental Clinic, Tokyo, Japan) and a private room in a university dental hospital (Nihon University Graduate School of Dentistry at Matsudo, Matsudo, Japan) were selected, and simulated scaling was performed by dentists and dental hygienists on three healthy volunteers (31~52 years old) two times in a private office and one time in an university dental hospital. Actual scaling was not performed, and, instead of actual scaling, simulated scaling (Scaler did not attach teeth and calculus) was performed. Extraoral suction (ARTEO, Tokyo Giken, INC., Tokyo, Japan) and oral suction attached to the dental chair were used to protect the spread of droplets and aerosols during dental treatment. The groups using extraoral and oral suctions were divided into 3 groups: A group: no oral suction and no extraoral suction, B group: oral suction and no extraoral suction, and C group: oral suction and extraoral suction. Simulated scaling was performed for 10 min, droplets and aerosols fell in open culture plates located around the dental chairs (Figure 1), and plates were closed by lids 10 min later after scaling was finished. Swab samples were taken by a LuciPac pen (Kikkoman Biochemifa Company, Tokyo, Japan) around the dental chair (Figure 2A,B). This simulated scaling and sampling around dental chair were performed on 30 July–8 October in 2020. This study was not submitted to the Ethics Commission because a similar study previously submitted to the Ethics Commission in National Institute of Infectious Diseases was not considered (project code: 424).

### 2.2. Measurement of Bacterial Numbers in Droplets after Scaling

Counting of colony forming units (CFU) of bacteria in droplets from the floor around the dental chair was performed on brain heart infusion (BHI) agar plates and R2A agar plates. One BHI agar and two R2A agar culture plates were placed radially from the dental chair at 0.5- or 1-m intervals (Figure 1). After opening of lids of plates, simulated scaling for 10 min was performed by right-handed dental hygienists and dentists to healthy volunteers. Lids were closed after waiting for 10 min and finish of scaling. Bacteria in droplets and aerosols were observed by incubation of BHI and R2A agar plates, respectively. BHI agar was incubated for 48 h at 37 °C, and two R2A agars were incubated for 96~120 h at 25 °C and 35 °C. After incubation, colony numbers were counted.

### 2.3. Measurements of ATP in Droplets and Aerosols after Scaling

Measurements of droplets and aerosols were indirectly performed using quantitative analysis of adenosine triphosphate (ATP) in swab samples on surfaces around dental chairs (Figure 2A,B) that were produced in treatment by an ultrasonic scaler. To scientifically assess the effects of extraoral suction on the droplets produced by treatments using an ultrasonic scaler, a private dental clinic and university dental hospital provided dental chair units in private rooms for research. Simulated scaling for 10 min was performed by right-handed dental hygienists and dentists in healthy volunteers. Samples were swabbed and taken by a LuciPac pen (Kikkoman Biochemicfa Company, Tokyo, Japan) after waiting for 10 min and scaling. ATPs in swab samples were reacted with the luciferin/luciferase compounds present around the swab to produce bioluminescent light in a luminometer (Lumitester, Kikkoman), respectively. The No. 1 (behind left desk), No. 2 (bottom of bracket table), No. 3 (scaler tube surface), No. 4 (bracket table top button), No. 5 (left desk), No. 6 (front wall), No. 7 (left extraoral suction arm), No. 8 (dental chair shoulder), No. 9 (dental chair holding side), No. 10 (monitor), No. 11 (right extraoral suction arm tip), No. 12 (right extraoral suction arm tip), No. 13 (dental spittoon side), and No. 14 (right sidewall) sampling positions were swabbed by a LuciPac pen to measure ATP in droplets and aerosols (Figure 2A,B). ATP is the molecule used for energy storage by all types of living cells (animal, plant, bacterial, yeast, and mold). ATP transfers energy within living cells and supplies the enzymes necessary for cellular functions. After cell death, ATP is degraded by autolysis within minutes. Droplets and aerosols are measured by the luminescence reaction of fireflies to the amount of ATP contained in them. The firefly has two chemical compounds that react with ATP to produce bioluminescent light, luciferin and luciferase. The amount of bioluminescent light was measured by a luminometer and is expressed in relative light units (RLUs). The RLU varies are directly proportional to the amount of ATP from the microorganisms and therefore comparable to the amount of microbial biomass on the sampled surface around the dental chair. Initial cleanliness threshold is less than 500 RLU [20].

### 2.4. Statistical Analysis

The RLU and CFU levels are expressed as the mean ± standard deviation. All experiments were repeated independently three times. The statistical significance of differences among the A, B and C groups was determined using a one-way analysis of variance (ANOVA) and Kruskal–Wallis test (IBM SPSS statistics 24, IBM Corporation, Armonk, NY, USA). Differences between two groups were determined using a post hoc test (Bonferroni, IBM SPSS statistics 24). A *p*-value less than 0.05 was considered significant.

## 3. Results

**Figure 3:**
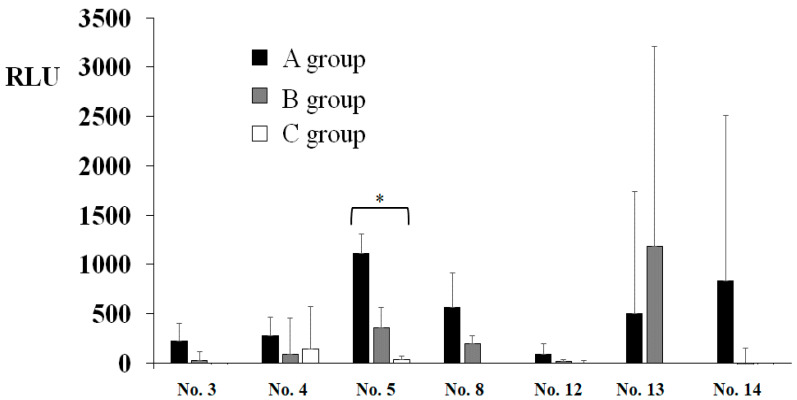
Levels of RLU in droplets and aerosols in various areas around the dental chair. Areas positively detected in A group (None) were selected, and the effects of B group (oral suction), and C group (extraoral and oral suction) on the levels of RLU that indicate luminous activity of ATP to luciferin/luciferase are presented. Locations in No. 3–5, No. 8, and No. 12–14 were indicated in Figure 2. The data indicate the mean ± standard deviation (SD) of three independent experiments. An asterisk indicates a significant difference among the three groups (*: *p* < 0.05, A–C).

**Figure 4:**
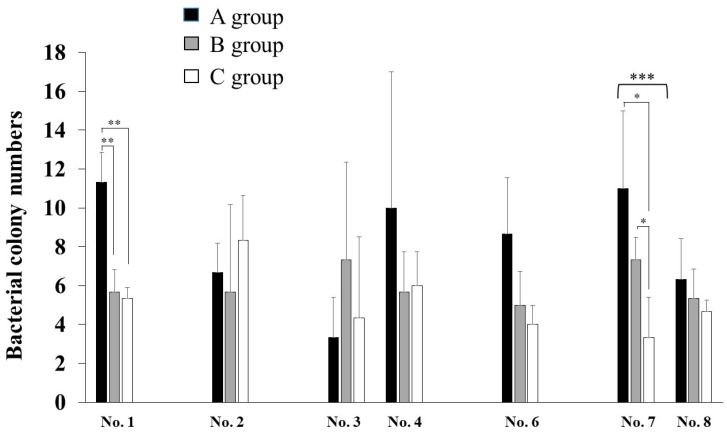
Bacterial numbers in droplets and aerosols that fall around the dental chair. Areas positively detected in the A group (None) were selected, and the effects of the B group (oral suction) and the C group (extraoral and oral suctions) on the bacterial numbers grown on BUI and R2A agar plates are presented. Locations in No. 1–4 and No. 6–8 were indicated in Figure 1. The data indicate the mean ± standard deviation (SD) of three independent experiments. The asterisks indicate a significant difference between two groups (*: *p* < 0.05, A vs. C, and B vs. C) and (**: *p* < 0.05, A vs. B, and A vs. C), and among the three groups (***: *p* < 0.05, A–C).

To assess the effects of extraoral suction on the spread of infection risk in dental treatment, ATP and colonization of bacteria in droplets and aerosols were observed. A significantly higher level of RLU was observed to an initial cleanliness threshold (less than 500 RLU) in only the No. 5 sample (Figure 3). The other samples did not show significant values to an initial cleanliness threshold. The RLU values in the samples from No. 1, No. 2, No. 6, No. 7, No. 9, No. 10, and No. 11 were too low, so the data were excluded. As a result of three groups comparison, the RLU levels were significantly lower in the oral suction only group (B) and the oral and extraoral suction groups (C) than in the no suction group (A) in only the No. 5 sample, located on the left desk to dental chair head (Figure 2B), by ANOVA analysis (Figure 3). The C group showed more inhibition effects than the B group, but there was no significant difference between the B and C groups. On other samples, there were no significant differences among groups. Bacterial colonies on the BHI and R2A agar plates were counted in droplets, and aerosols fell on the various areas after the treatment (Figure 1). The highest levels of colony numbers in A group that did not perform oral and extraoral suctions were around 10 in samples from No. 1, No. 4, and No. 7 (Figure 4). In contrast, data in sample from No. 5 did not show significant increase of colonies and, therefore, was excluded. As a result of three groups comparison, the colony numbers on R2A agar plates were significantly lower in the B group and C group than in the A group in the No. 7 samples by ANOVA analysis (Figure 4). In the relative comparison between two groups, the colony numbers in No. 1, located behind the dental chair head, were significantly lower in the B and C groups than in the A group by post hoc test (Figure 4). Therefore, this suggested that oral suction was effective for the reduction of droplets and aerosols. The colony numbers in area No. 7, located left-behind the dental chair head, were significantly lower in C group than in A group or B group by post hoc test (Figure 4). Therefore, this suggested that extraoral suction was effective for the reduction of droplets and aerosols as compared with oral suction and no suctions. On other samples, there were no significant differences. 

## 4. Discussion

The ATP and bacteria in droplets and aerosols produced during simulated scaling were quantitatively observed by reactions with luciferin/luciferase and incubation in culture plates to grow bacteria, respectively. The inhibition effects of oral and extraoral suctions were revealed on RLU in sample No. 5 at the left desk, and colonies in sample No. 1 at behind the dental chair head, and sample No. 7 at left-behind dental chair head. The protection against spreading droplets and aerosols by the combination of oral and extraoral suction was recognized, and the protected areas were limited to the left and posterior sides of the dental chair head when a right-handed dentist and dental hygienist performed scaling. In particular, the extraoral suction was effective for reducing droplets and aerosols in the limited area of the left side. While a right-handed dentist is treating a patient, a dental assistant often stands to the left to support the dentist. Therefore, the risk of infection for dental assistants is high. The extraoral suction may further reduce the risk of infection for dental assistants in addition to dentists and patients. 

Colony numbers were significantly counted in R2A. In contrast, there were no significant colony numbers on the BHI agar plates (data not shown). Oral bacteria were usually grown on BHI agar plates, and heterotrophic bacteria in water were grown on R2A agar plates. These waters in three times dental chairs were included less than 500 CFU/mL of heterotrophic bacteria on R2A. It was considered that this simulated scaling mainly produced droplets and aerosols by water from the water line on an ultrasonic scaler compared with saliva. However, a small amount of saliva is also contained in the droplets and aerosols. There is still a risk of infection in the droplets and aerosols. 

Taken together, the combination of oral and extraoral suction was effective for protecting against spreading droplets and aerosols, including large volumes of water and small volumes of saliva from the treatment sides to the left and posterior sides. In particular, protective effects of extraoral suction were revealed on the infection operator and for treatment assistance. Attitudes and knowledge for the prevention of nosocomial infection and compliance with ICPs are still insufficient in many countries worldwide [21,22,23,24]. Japanese dentists who were ready for infection control and extraoral suction might be able to deal with any infectious diseases. This study is very useful for understanding the infection risk area in COVID-19 pandemic [2,3,4,5,25,26] and effects of ICPs involving extraoral suction on the spread of pathogens such as viruses and bacteria in dental treatments. Therefore, the high standards of ICPs involving extraoral suction in dentists may help to reduce the risk of COVID-19 in dental care worldwide.

## 5. Conclusions

Regular dental treatments using the high-speed turbine handpiece and the ultrasonic scalers produce droplets and aerosols, providing possible hazards for dental staff and patients. Therefore, SARS-CoV-2 transmission during dental procedures can occur through inhalation of droplets and aerosols from infected individuals or direct contact with mucous membranes, oral fluids, and contaminated instruments and surfaces. Extraoral suction is a very useful tool for reducing production of droplets and aerosols that are restricted to the left and posterior sides of the dental chair head when a right-handed operators perform dental treatments. 

## Data Availability

Not applicable.
